# Sorry, Am I Intruding? Comparing Performance and Intrusion Rates for Pretested and Posttested Information

**DOI:** 10.3390/bs15081060

**Published:** 2025-08-05

**Authors:** Kelsey K. James, Benjamin C. Storm

**Affiliations:** 1 Department of Psychology, University of Houston Clear Lake, Houston, TX 77058, USA; 2 Department of Psychology, University of California, Santa Cruz, CA 95064, USA; storm@ucsc.edu

**Keywords:** pretesting, the pretesting effect, testing, the testing effect, true/false testing, learning, errors, the negative suggestion effect

## Abstract

Pretesting and posttesting have long been implemented in classrooms as methods of testing and improving learning. Prior research has been mixed on the relative benefits of pretesting versus posttesting, with some studies finding pretesting to be more beneficial, and others finding posttesting to be more beneficial. True/False testing is a particularly easy-to-implement method and is regularly used in classrooms. However, relatively little is known about how these tests affect learning. Three experiments address the effects of true/false pre- and posttests on learning correct information and intrusion rates of false information. We find consistent benefits of both pretesting and posttesting but significantly higher intrusion rates for posttesting relative to pretesting, a finding that persisted despite inclusion of simple True/False feedback (Experiment 2) and substantive feedback (Experiment 3). Although the difference between pretesting and posttesting intrusion rates was still significant with the addition of substantive feedback, overall intrusion rates were greatly reduced.

## 1. Introduction

### 1.1. Pretesting and Posttesting

The benefits of taking tests after learning are well established and have been demonstrated repeatedly across a multitude of factors, including learning media, testing format, and retention intervals (e.g., Chan & McDermott, 2007; Cranney et al., 2009; Little & Bjork, 2015; Roediger & Karpicke, 2006; Roediger et al., 2011a, 2011b; Rowland, 2014; Spitzer, 1939). In typical studies on the testing effect (hereafter referred to as the posttesting effect), participants study information (e.g., trivia, word pairs, lectures, or passages) and are then tested on a subset of that information before being tested again, often after a delay. Information initially tested via posttest tends to be significantly better recalled at final test than information either not initially tested or simply re-studied, an effect that has been observed in many conditions and with a variety of to-be-learned materials.

Research has shown, however, that taking tests before studying to-be-learned material can also benefit learning, a phenomenon often referred to as the pretesting effect (e.g., Carpenter & Toftness, 2017; Grimaldi & Karpicke, 2012; James & Storm, 2019; Kornell, 2014; Kriechbaum & Bäuml, 2024; Little & Bjork, 2016; Overoye et al., 2021; Pastötter & Bäuml, 2014; Richland et al., 2009). In typical studies on the pretesting effect, participants begin by taking a test on information they have not yet studied. After completing this pretest (usually with little success), participants are presented with the information needed to answer the questions from the pretest. Similar to posttesting, participants are then given a final test, usually after a delay, and on this final test, participants perform better on pretested information than on non-pretested information.

Although both pretesting and posttesting can be beneficial, their relative effectiveness remains unclear. Some studies have observed a greater benefit of posttesting than pretesting (Geller et al., 2017; Latimier et al., 2019; McDaniel et al., 2011), whereas others have found the opposite (Pan & Sana, 2021). There are compelling reasons to expect posttesting to be more effective. For example, posttesting occurs when learners have already been exposed to the material, a factor which can facilitate retrieval practice performance and make it easier for learners to incorporate new information into memory. In contrast, learners may be unable to answer any questions correctly on a pretest, as they have not yet studied the to-be-learned material. On the other hand, pretesting has the advantage of allowing learners to study the to-be-learned information after taking the pretest, which may focus encoding efforts on that specific information. Indeed, the study phase that follows a pretest can provide direct, powerful feedback, allowing learners to correct errors and solidify knowledge, a feature less likely to take place in the case of posttesting.

### 1.2. True/False Testing

True/False tests are frequently used in educational settings due to their simplicity and practicality. They are easy to create and grade and can be incorporated into other forms of assessment such as multiple-choice scantron tests. Despite their frequent use, relatively little is known about how they impact learning and whether and to what extent they lead to the kind of pretesting and posttesting benefits described above. There do seem to be benefits, albeit somewhat more mixed compared to what has been observed with other kinds of tests. In one study, for example, Uner et al. (2022) found a posttesting benefit for True/False quizzed items when compared to a re-reading control but not a re-typing control. In another study, Brabec et al. (2021) sought to determine the benefits of True/False posttesting on both tested information and nontested, related information. Interestingly, they found a benefit for tested information when the correct answer on the posttest was “true” and a benefit for nontested information when the correct answer on the posttest was “false.” In other recent work on True/False testing, Hildenbrand and Wiley (2025) demonstrated benefits for True/False testing when the final test was an essay (Exp. 1) but not when it was short answer (Exp. 2). Subsequent experiments showed that multiple-choice tests led to better short answer final test performance than True/False tests regardless of feedback (Exp. 3) and text availability (Exp. 4).

An important limitation of True/False testing is that, by the nature of the test, false information is presented to the learners. According to the negative suggestion effect, which was first discussed by Remmers and Remmers (1926), exposure to false information—such as in the context of false items in a True/False test—can lead to worse memory for correct information and a higher likelihood of endorsing false information as true. While Remmers and Remmers did not actually find evidence of a negative suggestion effect in their study, another study by McClusky (1934) found it shortly thereafter, and several other more recent studies found evidence of a negative suggestion effect as well (e.g., Toppino & Brochin, 1989; Toppino & Luipersbeck, 1993). Additionally, related work on multiple-choice testing (Roediger & Marsh, 2005) has shown that multiple-choice tests can lead to a negative suggestion effect, with learners subsequently endorsing incorrect alternatives.

### 1.3. Pretesting and Posttesting with True/False Tests

The negative suggestion effect from the false items of True/False tests presents a unique opportunity that, to our knowledge, has not been explored in prior research. Specifically, we compare the negative suggestion effect between pretesting and posttesting. Is one kind of test more likely to lead to a negative suggestion effect than the other? We sought to answer this question by measuring the rate of “intrusions” at final test (i.e., the probability of incorrectly recalling a false item that was used in a pre/post TF test).

There are reasons to think that pretesting should lead to more false intrusions than posttesting. Exposure to false information prior to studying true information may leave participants vulnerable to incorporating the false information into existing knowledge structures, thereby making participants more likely to output the false information when tested. When taking a posttest, participants have the benefit of having already studied the true information, putting them in a better position to protect themselves from incorporating the false information. That said, there are reasons to think participants will be less susceptible to false intrusions following pretesting than posttesting. When engaged in pretesting, participants view the information they encounter with more uncertainty, perhaps making them resistant to encoding that information in a way that makes it memorable at final test. Participants may also more effectively discount information that comes to mind when recalled as having been encountered during pretesting, as they would know by virtue of that context that they should be uncertain about its veracity. Finally, participants may activate details from the text when taking a posttest, thereby leading to subsequent confusion at the time of the final test, a factor that would not be relevant in the context of pretesting.

### 1.4. The Present Experiments

The aim of the present experiments was to examine the benefits and limitations of pretesting and posttesting in the context of True/False questions. More specifically, the goals were to determine (1) the degree to which the benefits of pretesting and posttesting hold up with True/False tests, (2) the relative effectiveness of each method for promoting performance on a final test, and (3) the relative extent to which each test type leads to a negative suggestion effect in the form of intrusions from false information.

To preview our results, no significant differences were found between overall final test performance following pretests and posttests. However, a significantly greater number of false intrusions were found following posttests than pretests. This result was found without feedback (Experiment 1), with simple corrective feedback (just that the answer was true/false; Experiment 2), and with substantive feedback (with the statement repeated with the true information following the guess; Experiment 3), though the difference was much smaller and overall intrusion rates were much lower in the final case.

## 2. Experiment 1

The primary goal of the first experiment was to compare pretesting and posttesting in the context of true/false test-taking. We sought to compare the benefits of taking such tests not only on their potential to improve final test performance, but on their susceptibility to negative suggestion effects at final test in the form of intrusions from false information presented during pretesting/posttesting. We also examined whether such effects depend on the nature of the true/false items, such as whether the correct answer on a given test was true or false.

### 2.1. Methods

#### 2.1.1. Participants and Design

A total of 128 undergraduates (*M* age: 20.2 years, range = 18–28; 93 women, 31 men, 2 write-in responses, 2 declined to state) at the University of California, Santa Cruz (UCSC), participated for partial credit in a psychology course as approved by the university’s IRB. The sample size was determined prior to beginning data collection. Specifically, we aimed to have enough participants to provide 80% power to observe a relatively small effect size (*d* = 0.25) when comparing performance and intrusion rates in the Pretest and Posttest conditions. A 2 (Test Type: Pretest vs. Posttest) × 3 (Item Type: True-Tested, False-Tested, Untested) within-subjects design was used. Counterbalancing was implemented such that half the participants received a pretest for the first passage and a posttest for the second passage, and the other half vice versa. Counterbalancing across participants also ensured that each to-be-learned fact was equally likely to be pretested, posttested, or untested and, if tested, whether the statement presented was true or false. For clarity in our analyses, we use the term Question Type to refer to whether a given statement was presented as true or false during the pre/posttest, and Item Type to refer to the learning history of a given fact on the final test (True-Tested, False-Tested, or Untested).

#### 2.1.2. Materials

Two passages were selected from James and Storm (2019): one focused on Joan of Arc (910 words) and the other on Machu Picchu (824 words). For each passage, we created a set of 16 critical facts. To implement our counterbalancing scheme, the 16 facts were distributed across four unique test lists. On a given list, 8 of the 16 facts were assigned to be tested and the other 8 were untested. Of the 8 tested facts, 4 were presented as true statements and 4 were presented as false statements. Across the four lists, each of the 16 critical facts appeared once as a true-tested item, once as a false-tested item, and twice as an untested item, ensuring complete counterbalancing of items across conditions. Each participant was randomly assigned to one of the four lists for their pretest passage and to one of the four lists for their posttest passage. For a given tested item, a participant was shown either the true statement or the false statement, but not both. False items were created with the goal of still making sense within the item with only one word or short phrase changed as compared to the true item.

Final tests consisted of all 16 questions from each passage in cued-recall form. The cued recall items were created either by removing the true/false piece of information (a single word or short phrase) from the initial question and leaving it as a blank or by reframing the initial test item into a question with the answer being the true/false piece of information. For example, the initial T/F item “As part of her mission, Joan of Arc took a vow of chastity; As part of her mission, Joan of Arc took a vow of silence” (true and false, respectively), participants were tested on the cued-recall test with the question “As part of her mission, Joan of Arc took a vow of _________”, with the correct answer being “chastity”. For the final cued recall test, if a participant answered “chastity” or something with an equivalent meaning, e.g., “celibacy”, this would be coded as a “correct” answer, but if they answered with “silence” or something equivalent, this would be coded as an “intrusion” answer. Any other answer or leaving the item blank would be coded as “incorrect”. The full set of items, including true/false statements and cued-recall questions, can be found in Appendix A.

#### 2.1.3. Procedure

Participants were tested individually or in groups of up to 3. The experiment took place in person in the lab, with test questions presented over OpenSesame and with the passages administered on paper. The passages were always administered in the same order for every participant: Joan of Arc then Machu Picchu. Half of the participants received a Pretest for Joan of Arc and a Posttest for Machu Picchu, and half vice versa and participants were randomly assigned to one of these counterbalanced orders. Pretest and Posttest questions were administered one at a time with participants required to select True or False for each item before moving on to the next question. After completing both passages and their respective pre- or posttest, participants were given 5 min to play Sudoku. Then, participants completed a final test including all 32 cued recall items. All Joan of Arc test items were administered first, followed by all Machu Picchu items. Once again, the final test questions were presented one at a time with participants required to write something before moving on to the next question.

Final test answers were coded by the experimenter as either correct, an intrusion, or incorrect. Coding in this experiment, and in all subsequent experiments, was conducted by the first author, who was blind to condition. To be counted as an intrusion, participants needed to output the specific piece of information from the corresponding false item for that question. The coding was done in this way regardless of whether the specific participant had encountered that item as false (i.e., whether or not that participant had actually been exposed to that false information) in order to establish a baseline rate at which participants might output that specific answer. With the example used previously, “As part of her mission, Joan of Arc took a vow of _________”, the intrusion answer, “silence”, is something that it is not entirely unreasonable to guess based on prior knowledge even if a participant had not encountered it as part of their pre/posttest.

### 2.2. Results

#### 2.2.1. Pretest and Posttest Performance

**Figure 1:**
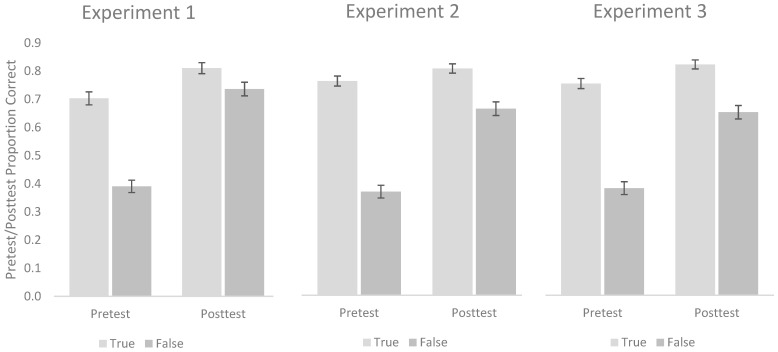
Pretest and posttest performance across the three experiments as a function of whether the correct answer on a given question was True or False. Error bars represent standard error of the mean.

We conducted a 2 (Test Type: Pretest vs. Posttest) by 2 (Question Type: True vs. False) repeated-measures ANOVA. As can be seen in the left panel of Figure 1, two main effects were observed such that performance on the posttest (*M* = 0.77, *SD* = 0.17) was significantly better than on the pretest (*M* = 0.55, *SD* = 0.19), *F*(1, 127) = 109.10, *p* < 0.001, *η*^2^*_p_* = 0.46, and performance on True Questions (*M* = 0.76, *SD* = 0.17) was significantly better than on False Questions (*M* = 0.56, *SD* = 0.19), *F*(1, 127) = 83.16, *p* < 0.001, *η*^2^*_p_* = 0.40. Moreover, a significant interaction was observed between Test Type and Question Type, *F*(1, 127) = 39.52, *p* < 0.001, *η*^2^*_p_* = 0.24. Follow-up tests showed that although performance was better on True questions than False questions on both the Pretest, *t*(127) = 11.27, *p* < 0.001, *d* = 1.00, 95% CI [0.78–1.20], BF_01_ < 0.001, and the Posttest, *t*(127) = 2.55, *p* = 0.01, *d* = 0.23, 95% CI [0.05–0.40], BF_01_ = 0.617, the difference was much greater on the pretest than on the posttest. 

The overall gap in performance between performance on the pretest and the posttest is to be expected given that participants had not yet been exposed to the information at the time of the pretest. It is notable, however, that we observed a true response bias in both the posttest and, to a much greater degree, the pretest. It seems that, with these questions, in the absence of additional information, learners err on the side of assuming information is correct. This fits with prior work demonstrating a “veracity bias” (Levine et al., 1999) in which people tend to assume information provided is true.

#### 2.2.2. Final Test Performance

**Figure 2:**
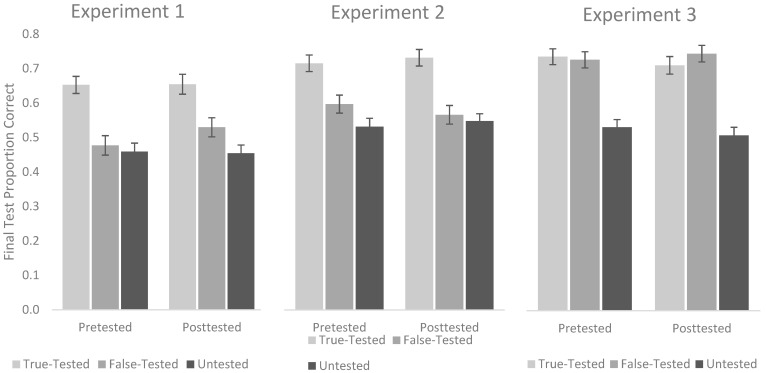
Final test performance across the three experiments as a function of Item Type and whether participants received a pretest or a posttest on a given passage. Error bars represent standard error of the mean.

We next compared the magnitude of the benefits of pretesting and posttesting by conducting a 2 (Test Type: Pretest vs. Posttest) by 3 (Item Type: True-Tested vs. False-Tested vs. Untested) repeated-measures ANOVA on final test performance. As can be seen in the left panel of Figure 2, a main effect of Test Type was not observed, *F*(1, 127) = 0.49, *p* = 0.47, *η*^2^*_p_* = 0.00. Specifically, final test performance did not differ depending on whether participants received a pretest (*M* = 0.53, *SD* = 0.22) or a posttest (*M* = 0.55, *SD* = 0.23). A main effect of Item Type was observed, *F*(2, 254) = 58.89, *p* < 0.001, *η*^2^*_p_* = 0.32, however, such that True-Tested items (*M* = 0.65, *SD* = 0.24) were recalled better than both False-Tested items (*M* = 0.50, *SD* = 0.23), *t*(127) = 7.73, *p* < 0.001, *d* = 0.68, 95% CI [0.49, 0.88], BF_01_ < 0.001, and Untested items (*M* = 0.46, *SD* = 0.20), *t*(127) = 10.01, *p* < 0.001, *d* = 0.88, 95% CI [0.68, 1.09], BF_01_ < 0.001. False-Tested items were also recalled better than Untested items, *t*(127) = 2.72, *p* = 0.007, *d* = 0.24, 95% CI [0.06, 0.42], BF_01_ = 0.405. Finally, we failed to observe a significant interaction, *F*(2, 254) = 0.93, *p* = 0.40, *η*^2^*_p_* = 0.01. Thus, we failed to find evidence that participants benefited from pretesting and posttesting differently as a function of Item Type.

#### 2.2.3. Final Test Intrusions

**Figure 3:**
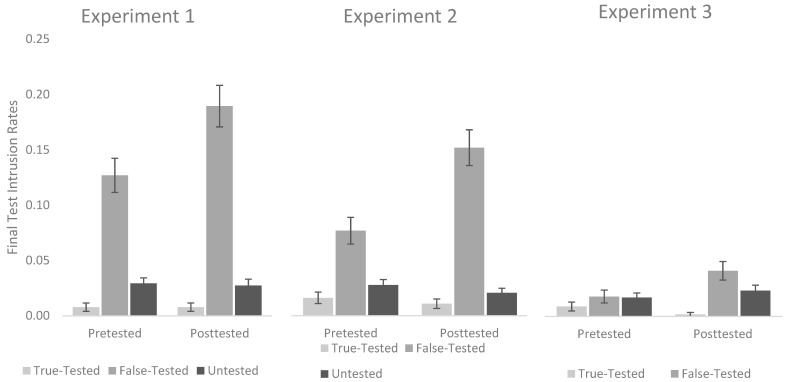
False intrusion rates across the three experiments as a function of Item Type and whether participants received a pretest or a posttest on a given passage. Error bars represent standard error of the mean.

To test our primary hypothesis regarding false intrusions, we conducted a 2 (Test Type: Pretested vs. Posttested) by 3 (Item Type: True-Tested vs. False-Tested vs. Untested) repeated-measures ANOVA on the proportion of intrusions on the final test. This analysis revealed significant main effects of both Item Type, *F*(2, 254) = 114.86, *p* < 0.001, *η*^2^*_p_* = 0.47, and Test Type, *F*(1, 127) = 5.13, *p* = 0.025, *η*^2^*_p_* = 0.04, which were in turn qualified by a significant interaction, *F*(2, 254) = 6.17, *p* = 0.002, *η*^2^*_p_* = 0.05. To decompose this interaction, we directly compared the intrusion rates for the critical False-Tested items across the two test types. As can be seen in the left panel of Figure 3, participants produced significantly more intrusions for items that were false on the Posttest (*M* = 0.19, *SD* = 0.21) than they did for items that were false on the Pretest (*M* = 0.13, *SD* = 0.17), *t*(127) = 2.60, *p* = 0.011, *d* = 0.23, 95% CI [0.05, 0.40], BF_01_ = 0.389.

Examining the simple effects of Item Type within each test condition revealed significant effects in both the Pretest condition, *F*(2, 254) = 44.80, *p* < 0.001, *η*^2^*_p_* = 0.26, and the Posttest condition, *F*(2, 254) = 76.78, *p* < 0.001, *η*^2^*_p_* = 0.38. Focusing first on the pretest condition, we found that the intrusion rate was significantly higher for False-Tested items (*M* = 0.13, *SD* = 0.17) than both Untested items (*M* = 0.03, *SD* = 0.06), *t*(127) = 6.40, *p* < 0.001, *d* = 0.57, 95% CI [0.38, 0.75], BF_01_ < 0.001, and True-Tested items (*M* = 0.01, *SD* = 0.04), *t*(127) = 7.27, *p* < 0.001, *d* = 0.64, 95% CI [0.45, 0.83], BF_01_ < 0.001, while being significantly lower for True-Tested items than Untested items, *t*(127) = 3.46, *p* < 0.001, *d* = 0.31, 95% CI [0.13, 0.48], BF_01_ = 0.050. A similar pattern was observed in the Posttest condition: the intrusion rate was significantly higher for False-Tested items (*M* = 0.19, *SD* = 0.21) than both Untested items (*M* = 0.03, *SD* = 0.06), *t*(127) = 8.45, *p* < 0.001, *d* = 0.75, 95% CI [0.55, 0.94], BF_01_ < 0.001, and True-Tested items (*M* = 0.01, *SD* = 0.04), *t*(127) = 9.56, *p* < 0.001, *d* = 0.85, 95% CI [0.64, 1.05], BF_01_ < 0.001, while being significantly lower for True-Tested items than Untested items, *t*(127) = 2.85, *p* = 0.005, *d* = 0.25, 95% CI [0.08, 0.43], BF_01_ = 0.287.

## 3. Experiment 2

In Experiment 1, no differences were found on final test performance between items that were pretested versus posttested. Specifically, both types of tests appeared to promote recall performance at final test, and to an extent that did not significantly differ. However, a significantly higher proportion of intrusions did result from taking a posttest than taking a pretest, with this finding being observed despite posttest performance being significantly better than pretest performance. The goal of experiment 2 was to replicate experiment 1 and to examine how the addition of corrective feedback affects final test performance and intrusion rates.

Feedback has been demonstrated to be important for both pretesting and posttesting, though it is unclear whether it is equally important. By the nature of pretesting, the correct information is encountered after the pretest, so even without feedback, the final information encountered prior to the final test is the correct information. With posttesting, however, the order is study then test. As a consequence, for any “False” item, the last statement a participant encounters will be incorrect. Recent exposure to misinformation may therefore make posttesting especially prone to creating false memories—a disadvantage that corrective feedback may be able to help overcome. Such a finding would be consistent with prior work showing that the addition of feedback strengthens the posttesting benefit (Butler & Roediger, 2008; Kang et al., 2007). Indeed, McClusky (1934) suggested feedback as an antidote to the negative suggestion effect found in his experiments with True/False tests.

### 3.1. Methods

#### 3.1.1. Participants and Design

A total of 140 UCSC undergraduates (*M* age: 19.86 years, range = 18–34; 110 female, 28 male, 1 write-in response, 1 declined to state) participated for partial credit in a psychology course as approved by the university’s IRB. The study design was identical to that of Experiment 1. A power analysis, based on the effect size for the difference in intrusion rates between pretested and posttested false items in Experiment 1 (*d* = 0.23), suggested that 135 participants would be necessary for the experiment to have 80% power to observe a similar effect size in Experiment 2. As in Experiment 1, the study was initially conducted in person using OpenSesame (*n* = 29). To expedite data collection, however, we transitioned to run all subsequent participants remotely using the online survey platform, Qualtrics (*n* = 111). With this shift, we changed the distractor task from Sudoku to a word search.

#### 3.1.2. Materials and Procedure

The materials and procedure for Experiment 2 were identical to that of Experiment 1, with the exception that corrective feedback was provided after each pre/posttest question. Specifically, after participants submitted their response, the statement “The correct answer is TRUE” or “The correct answer is FALSE” was displayed on screen until the participant pressed an arrow button to continue. This feedback was provided on every trial, regardless of the participant’s accuracy in responding to the pre/posttest.

### 3.2. Results

#### 3.2.1. Pretest and Posttest Performance

Similar to Experiment 1, we conducted a 2 (Test Type: Pretest vs. Posttest) by 2 (Question Type: True vs. False) repeated-measures ANOVA. As can be seen in the center panel of Figure 1, main effects were observed such that performance on the posttest (*M* = 0.73, *SD* = 0.19) was significantly higher than performance on the pretest (*M* = 0.56, *SD* = 0.20), *F*(1, 139) = 55.13, *p* < 0.001, *η*^2^*_p_* = 0.28., and performance on True Questions (*M* = 0.78, *SD* = 0.15) was significantly better than on False Questions (*M* = 0.52, *SD* = 0.20), *F*(1, 139) = 218.40, *p* < 0.001, *η*^2^*_p_* = 0.61. Moreover, a significant interaction was observed between Test Type and Question Type, *F*(1, 139) = 50.68, *p* < 0.001, *η*^2^*_p_* = 0.27. Follow-up tests revealed that although performance was better on True questions than False questions on both the pretest *t*(139) = 15.12, *p* < 0.001, *d* = 1.28, 95% CI [1.05–1.50], BF_01_ < 0.001, and posttest, *t*(139) = 5.84, *p* < 0.001, *d* = 0.49, 95% CI [0.32, 0.67], BF_01_ < 0.001, the difference was much greater on the pretest than on the posttest.

#### 3.2.2. Final Test Performance

We conducted a 2 (Test Type: Pretest vs. Posttest) by 3 (Item Type: True-Tested vs. False-Tested vs. Untested) repeated-measures ANOVA on the final test performance. As can be seen in the center panel of Figure 2, a main effect of Test Type was not observed, *F*(1, 139) = 0.00, *p* = 0.99, *η*^2^*_p_* = 0.00. Specifically, final test performance did not differ depending on whether participants received a pretest (*M* = 0.61, *SD* = 0.24) or a posttest, (*M* = 0.61, *SD* = 0.23). A main effect of Item Type was observed, *F*(2, 278) = 55.65, *p* < 0.001, *η*^2^*_p_* = 0.29, however, such that True-Tested Items (*M* = 0.72, *SD* = 0.24) were recalled better than both False-Tested Items (*M* = 0.58, *SD* = 0.26), *t*(139) = 6.93, *p* < 0.001, *d* = 0.59, 95% CI [0.41, 0.76], BF_01_ < 0.001, and Untested items (*M* = 0.54, *SD* = 0.22), *t*(139) = 10.55, *p* < 0.001, *d* = 0.89, 95% CI [0.69, 1.09], BF_01_ < 0.001. False-Tested items were also recalled better than Untested items, *t*(139) = 2.51, *p* = 0.013, *d* = 0.21, 95% CI [0.04, 0.38], BF_01_ = 0.880. Finally, we failed to detect a significant interaction, *F*(2, 278) = 1.19, *p* = 0.31, *η*^2^*_p_* = 0.01, and thus again failed to find evidence that performance on the pretest and posttest differed as a function of Item Type.

#### 3.2.3. Final Test Intrusions

We conducted a 2 (Test Type: Pretested vs. Posttested) by 3 (Item Type: True-Tested vs. False-Tested vs. Untested) repeated-measures ANOVA on the proportion of intrusions on the final test. As in Experiment 1, this analysis revealed significant main effects of both Item Type, *F*(2, 278) = 67.32, *p* < 0.001, *η*^2^*_p_* = 0.33, and Test Type, *F*(1, 139) = 10.29, *p* = 0.002, *η*^2^*_p_* = 0.07, which were in turn qualified by a significant interaction, *F*(2, 278) = 15.10, *p* < 0.001, *η*^2^*_p_* = 0.10. As can be seen in the center panel of Figure 3, when we compare the critical False-Tested items directly across the two test types, participants produced significantly more intrusions on items that were false on the posttest (*M* = 0.15, *SD* = 0.19) than they did for items that were false on the pretest (*M* = 0.08, *SD* = 0.06), *t*(139) = 4.04, *p* < 0.001, *d* = 0.34, 95% CI [0.17, 0.51], BF_01_ = 0.007.

Examining the simple effects of Item Type separately for each test condition revealed significant differences in both the Pretest condition, *F*(2, 278) = 15.97, *p* < 0.001, *η*^2^*_p_* = 0.10, and the Posttest condition, *F*(2, 278) = 62.61, *p* < 0.001, *η*^2^*_p_* = 0.31. Focusing first on the pretest condition, we found that the intrusion rate was significantly higher for False-Tested items (*M* = 0.08, *SD* = 0.06) than both Untested items (*M* = 0.03, *SD* = 0.06), *t*(139) = 3.70, *p* < 0.001, *d* = 0.31, 95% CI [0.14, 0.48], BF_01_ = 0.023, and True-Tested items (*M* = 0.02, *SD* = 0.06), *t*(139) = 4.71, *p* < 0.001, *d* = 0.40, 95% CI [0.23, 0.57], BF_01_ = 0.001. Unlike in Experiment 1, a significant difference was not observed between True-Tested items and Untested items, *t*(139) = 1.68, *p* = 0.096, *d* = 0.14, 95% CI [−0.03, 0.31], BF_01_ = 3.758. In the Posttest condition, the intrusion rate was significantly higher for False-Tested items (*M* = 0.15, *SD* = 0.19) than both Untested items (*M* = 0.02, *SD* = 0.05), *t*(139) = 7.82, *p* < 0.001, *d* = 0.66, 95% CI [0.48, 0.84], BF_01_ < 0.001, and True-Tested items (*M* = 0.01, *SD* = 0.05), *t*(139) = 8.37, *p* < 0.001 *d* = 0.71, 95% CI [0.52, 0.89], BF_01_ < 0.001, with once again a significant difference not being observed between True-Tested items and Untested items, items *t*(139) = 1.82, *p* = 0.07, *d* = 0.15, 95% CI [−0.01, 0.32], BF_01_ = 2.921.

## 4. Experiment 3

Overall, the patterns of results observed in Experiment 2 were largely the same as those observed in Experiment 1. Despite performance on the posttest being better than performance on the pretest, both tests led to comparable learning benefits relative to untested items, and both led to false intrusions. Most critically, posttesting led to more false intrusions than pretesting, with the effect size observed in Experiment 2 being just as large as it was in Experiment 1. Although corrective feedback did lead to lower intrusion rates overall (which can be seen visually in Figure 3), it did not appear to do so in a way that was more effective at reducing false intrusions following posttesting than after pretesting.

In Experiment 3 we examined whether more substantive feedback would be more effective in reducing the number of false intrusions resulting from posttests. Some prior research on errorful learning (e.g., Pashler et al., 2005) has demonstrated that more in-depth feedback, such as supplying answers rather than simple correct/incorrect feedback, may be critical for improving learning from errors. Whereas corrective feedback in Experiment 2 may have been effective at focusing attention towards the correct to-be-learned information following a pretest, it may not have been as effective following a posttest since there was not any opportunity to go back and study the to-be-learned information. When given substantive feedback, however, this difference may be mitigated. Providing learners with the correct to-be-learned information following a posttest may help reduce the likelihood that they will go on to intrude false information at final recall. Indeed, it may do so to such an extent that posttesting will no longer lead to rates of false intrusion greater than that observed following pretesting.

### 4.1. Methods

#### 4.1.1. Participants and Design

A total of 140 UCSC undergraduates (*M* age: 20.57 years, range = 18–50; 106 female, 27 male, 6 write-in responses, 1 declined to state) participated for partial credit in a psychology course as approved by the university’s IRB. The study design was identical to that of the first two experiments. Considering the similar structure to the previous experiments, we reused the power analysis from Experiment 2 with a goal of 135 participants. The experiment was run entirely online using Qualtrics.

#### 4.1.2. Materials and Procedure

The materials and procedure were identical to that of Experiment 2, with the exception that an additional sentence explaining the correct information was added to the feedback for each pre/posttest question. For example, after responding to a pre/posttest question such as “As part of her mission, Joan of Arc took a vow of silence.” the participant would receive the feedback: “The correct answer is FALSE; Joan took a vow of *chastity*.” For true items, the feedback similarly restated the correct fact. This additional sentence was added to the feedback regardless of whether the answer was true or false and regardless of whether the participant responded correctly. See the rightmost column of Appendix A for a full list of the feedback statements for each item.

### 4.2. Results

#### 4.2.1. Pretest and Posttest Performance

As in the prior experiments, we began by conducting a 2 (Test Type: Pretest vs. Posttest) by 2 (Question Type: True vs. False) repeated-measures ANOVA. As can be seen in the right panel of Figure 1, main effects were observed such that performance on the posttest (*M* = 0.74, *SD* = 0.19) was significantly better than on the pretest (*M* = 0.57, *SD* = 0.18), *F*(1, 139) = 59.72, *p* < 0.001, *η*^2^*_p_* = 0.30, and performance on the True Questions (*M* = 0.79, *SD* = 0.15) was significantly better than performance on the False Questions (*M* = 0.52, *SD* = 0.20), *F*(1, 139) = 202.91, *p* < 0.001, *η*^2^*_p_* = 0.59. A significant interaction was also found between Test Type and Question Type, *F*(1, 139) = 31.55, *p* < 0.001, *η*^2^*_p_* = 0.19. Follow up tests revealed once again that although performance was greater on True questions than False questions on both the pretest *t*(139) = 14.55, *p* < 0.001, *d* = 1.23, 95% CI [1.01–1.45], BF_01_ < 0.001, and the posttest, *t*(139) = 6.35, *p* < 0.001, *d* = 0.54, 95% CI [0.36, 0.71], BF_01_ < 0.001, the difference was much greater on the pretest than on the posttest.

#### 4.2.2. Final Test Performance

We conducted a 2 (Test Type: Pretest vs. Posttest) by 3 (Item Type: True-Tested vs. False-Tested vs. Untested) repeated-measures ANOVA on final test performance. As in the prior experiments, a main effect of Test Type was not observed, *F*(1, 139) = 0.35, *p* = 0.55, *η*^2^*_p_* = 0.00, with final test performance failing to differ as a function of whether participants received a pretest (*M* = 0.67, *SD* = 0.21) or a posttest (*M* = 0.66, *SD* = 0.23). A main effect of Item Type was observed, *F*(2, 278) = 81.01, *p* < 0.001, *η*^2^*_p_* = 0.37. Interestingly, True-Tested items (*M* = 0.72, *SD* = 0.25) were recalled better than Untested items (*M* = 0.52, *SD* = 0.21), *t*(139) = 10.43, *p* < 0.001, *d* = 0.88, 95% CI [0.69, 1.08], BF_01_ < 0.001, but they were not recalled better than False-Tested items (*M* = 0.74, *SD* = 0.23), *t*(139) = 0.64, *p* = 0.520, *d* = 0.05, 95% CI [−0.11, 0.22], BF_01_ = 12.154. False-Tested items were recalled better than Untested items, *t*(139) = 11.82, *p* < 0.001, *d* = 1.00, 95% CI [0.79, 1.20], BF_01_ < 0.001. Finally, we once again failed to observe a significant interaction, *F*(2, 278) = 1.08, *p* = 0.34, *η*^2^*_p_* = 0.01, and thus again failed to find evidence that performance on the pretest and posttest differed as a function of Item Type. Final test performance for Experiment 3 can be seen in the right panel of Figure 2.

#### 4.2.3. Final Test Intrusions

As in prior experiments, we conducted a 2 (Test Type: Pretested vs. Posttested) by 3 (Item Type: True-Tested vs. False-Tested vs. Untested) repeated-measures ANOVA on the proportion of intrusions on the final test. This analysis revealed a significant main effect of Item Type, *F*(2, 278) = 12.32, *p* < 0.001, *η*^2^*_p_* = 0.08, but not of Test Type, *F*(1, 139) = 3.25, *p* = 0.074, *η*^2^*_p_* = 0.02. Most importantly, a significant interaction was found between Test Type and Item Type, *F*(2, 278) = 4.15, *p* = 0.017, *η*^2^*_p_* = 0.03. As in the previous experiments, we began by comparing the critical False-Tested items directly across the two test types. Once again, participants produced significantly more intrusions on items that were false on the Posttest (*M* = 0.04, *SD* = 0.10) than they did for items that were false on the Pretest (*M* = 0.02, *SD* = 0.07), *t*(139) = 2.23, *p* = 0.027, *d* = 0.19, 95% CI [0.02, 0.36], BF_01_ = 1.324. As can be seen in the right panel of Figure 3, however, overall intrusion rates in Experiment 3 were substantially lower than in the previous experiments. It is worth noting that despite reaching the threshold for statistical significance using null-hypothesis testing, the Bayes Factor actually suggested nominally more evidence for the null. As a follow-up analysis, we combined data from all three experiments, and with this more highly powered comparison, we found strong evidence that participants produced significantly more intrusions on items that were false on the Posttest (*M* = 0.13, *SD* = 0.18) than they did for items that were false on the Pretest (*M* = 0.07, *SD* = 0.07), *t*(407) = 5.19, *p* < 0.001, *d* = 0.26, 95% CI [0.16, 0.36], BF_01_ < 0.001.

Examining the simple effects of Item Type separately for each test condition revealed significant differences in the posttest condition, *F*(2, 278) = 12.81, *p* < 0.001, *η*^2^*_p_* = 0.08, but not in the pretest condition, *F*(2, 278) = 0.98, *p* = 0.378, *η*^2^*_p_* = 0.01. This pattern suggests that the interaction was driven by substantive feedback being successful at eliminating the negative suggestion effective in the pretest condition, but not in the posttest condition. Indeed, the substantive feedback condition was so effective that it completely eliminated any significant differences in intrusion rates; intrusions for False-Tested items (*M* = 0.02, *SD* = 0.07) were reduced to the same floor-level as True-Tested items (*M* = 0.01, *SD* = 0.05) and Untested items (*M* = 0.02, *SD* = 0.04). Although dramatically reduced as well, small but significant differences remained in the posttest condition. Specifically, intrusion rates were still higher for False-Tested items (*M* = 0.04, *SD* = 0.10) than they were for either True-Tested items (*M* = 0.00, *SD* = 0.02), *t*(139) = 4.62, *p* < 0.001, *d* = 0.39, 95% CI [0.22, 0.56], BF_01_ = 0.001, or Untested items (*M* = 0.02, *SD* = 0.05), *t*(139) = 2.23, *p* = 0.027, *d* = 0.19, 95% CI [0.02, 0.36], BF_01_ = 1.307. Significantly fewer intrusions were observed on the True-Tested items than on the Untested items, *t*(139) = 3.83, *p* < 0.001, *d* = 0.32, 95% CI [0.15, 0.49], BF_01_ = 0.015.

## 5. General Discussion

Pretesting and posttesting have both been shown to be effective methods of improving final test performance (Karpicke & Roediger, 2008; Richland et al., 2009). Although many studies have focused on the benefits of either pretesting or posttesting alone, there have been relatively few comparing the two directly under the same set of conditions (Geller et al., 2017; McDaniel et al., 2011; Latimier et al., 2019; Pan & Sana, 2021). The present experiments add to this body of research by comparing pretesting and posttesting through the lens of True/False tests. In addition to examining the relative effectiveness of pretesting or posttesting for promoting subsequent recall performance, we also examined intrusion rates. Intrinsic to the nature of True/False tests is that test-takers will be exposed to false information. We sought to examine the extent to which such exposure leads to a negative suggestion effect (e.g., Remmers & Remmers, 1926) and whether this effect would be larger in the case of pretesting or posttesting.

In three experiments, participants read two educational passages, with one passage being preceded by a pretest, and the other being followed by a posttest. The pretest and posttest each consisted of a True/False test in which some items were true and other items were false. Not surprisingly, participants performed better on the posttest than they did on the pretest, a finding which makes sense given that participants had the opportunity to study the passage before taking the posttest. Nevertheless, when participants were given a final cued recall test, performance failed to differ as a function of test type, with both significantly enhancing performance relative to untested items. A significant difference was observed, however, in terms of intrusions. Specifically, posttesting led to significantly more intrusions than pretesting, with participants recalling the false information they were exposed to in the posttest significantly more often than the false information they were exposed to in the pretest. This difference was observed when participants were not given any feedback on the True/False tests (Experiment 1), when participants were given corrective feedback (Experiment 2), and when participants were given substantive feedback (Experiment 3). In fact, although substantive feedback effectively eliminated the negative suggestion following pretesting, it did not do so following posttesting.

A key finding from our experiments was that final test performance failed to differ as a function of pretesting and posttesting. This result is particularly interesting as it stands in contrast to other investigations, which have found either a pretesting advantage (e.g., Pan & Sana, 2021) or a posttesting advantage (e.g., McDaniel et al., 2011). One interpretation is that the relative efficacy of pretesting and posttesting is not absolute but rather depends on specific methodological factors, such as the format of the test questions, the extent to which pretesting enhances subsequent study behaviors, and the relative success of retrieval practice in posttesting. A pretesting advantage, for example, might emerge when test formats prompt deeper or more elaborative encoding during study, whereas a posttesting advantage may appear when conditions favor the successful retrieval practice and consolidation of recently learned material. Given the number of potential moderators, we are hesitant to overinterpret our null results. What our findings do suggest is that at least under the conditions we employed, taking a True/False test has the power to enhance learning, and it can do so regardless of whether it is administered before or after studying a to-be-learned passage.

It is noteworthy that learning from initially false statements was significantly less effective than learning from initially true statements. Across Experiments 1 and 2, while testing with true items provided a robust benefit to final performance, the benefit from testing with false items was varied and more limited. This pattern of results presents somewhat of a pedagogical challenge. Presumably, if a test consisted of only true items, the learning benefits from such items would be diminished, since learners would not have to think carefully about whether a given statement was true or false. Critically, in Experiment 3, the false items were found to benefit substantially from True/False testing, and to an extent that did not differ from that of true items. This finding suggests that all items have the potential to benefit from True/False testing as long as substantive feedback is provided.

Looking beyond overall test performance, our results demonstrate that taking a True/False test can put learners at risk for a negative suggestion effect. Specifically, exposure to incorrect information on a True/False test can make participants more likely to recall that information on the final test. This finding extends prior research demonstrating that exposure to incorrect lures on multiple-choice tests can increase their subsequent endorsement (e.g., Roediger & Marsh, 2005). Moreover, in the case of posttesting, a negative suggestion effect was observed even when participants were given substantive feedback following the True/False test. Specifically, participants continued to output the false information even when they were told that the information was false and given the correct information prior to taking the final test, albeit at a relatively low rate. The more important observation for present purposes was that across all three experiments, the negative suggestion effect was significantly larger following a True/False posttest than following a True/False pretest. Participants were consistently more likely to intrude incorrect information learned from exposure to false items on a posttest than they were from false items on a pretest, with this difference emerging even though participants were less likely to select incorrect information on the posttests than the pretests, and even though (as discussed above) posttesting led to overall final test performance that was not significantly different than pretesting. This pattern of results raises a critical theoretical question: what cognitive mechanisms make posttesting particularly vulnerable to the negative suggestion effect?

One plausible explanation centers on the recency of the misinformation. In Experiment 1, for example, because no feedback was given, the false statement on a posttest was the last piece of related information a participant encountered, which could have made it more accessible at the final test. This simple recency account, however, is challenged by the results of Experiment 3. In that experiment, substantive feedback presented the correct information after the false statement, yet the posttest intrusion rate remained higher than the pretest intrusion rate. Although recency may play a role, it seems unlikely to be the sole explanation.

A more convincing explanation may lie in the kind of cognitive mindset fostered by the two procedures. When taking a pretest, participants may undertake the pretest with a more evaluative or exploratory mindset. In this state, they may process the True/False questions critically, searching for clues and treating the information with a degree of skepticism that inoculates them against encoding the incorrect information in a way that would make that information likely to be retrieved as correct at final test. In contrast, because posttesting occurs after study, a learner’s goal is to try to retrieve accurate information and to do so in a way that is integrated with other information and knowledge about the topic. This could lead to higher intrusion rates by making false information more likely to be integrated into long-term memory.

Moreover, from a source monitoring perspective, participants may be better able to take advantage of context cues following pretesting than posttesting. In the case of pretesting, even if participants remember selecting a statement as being True, they can infer that because they made that selection prior to studying the to-be-learned passage, they should approach the veracity of that statement with some caution. In the case of posttesting, however, participants do not have this same benefit. If they remember selecting a given statement as True, they cannot use the general placement of the True/False test as an indication of the extent to which they were making that selection based on a guess or their memory for the information they had just studied. Indeed, posttesting may generally lead to more source confusion than pretesting, especially if participants are activating information from the passage while taking the posttest.

Finally, posttesting may lead to more intrusions than pretesting owing to dynamics related to retrieval-induced forgetting. Research has shown that retrieving some information can cause the forgetting of other information (Anderson et al., 1994; Bäuml & Kliegl, 2017; Storm & Levy, 2012). In the current context, a posttest TF false item may serve as a retrieval practice event, prompting the forgetting of the related true information. Consequently, when given the cued recall final test, participants may have been less likely to recall the true information, and therefore more likely to output the false information for those questions than they would have been otherwise. One might note that final recall performance of true information was not impaired by exposure to false TF items, but this observation does not negate the possibility that at least some of the true information experienced retrieval-induced forgetting. It is possible, for example, that whereas some true information benefited from posttesting via retrieval-induced facilitation (Bäuml & Samenieh, 2012; Chan et al., 2006; Oliva & Storm, 2023), other true information was impaired, and it was this latter case that contributed to the increase in false intrusions. Unfortunately, given the way the study was designed, it is impossible to separate the effects of posttesting on memory for false information from that of true information, but this is something future research may consider addressing more directly.

From a more applied perspective, the current results suggest that educators have good reason to be concerned about the negative suggestion effect in the context of True/False tests, especially when learners are not given feedback. In Experiment 1, participants recalled the False information 19% of the time, an intrusion rate that was lowered to 13% and 4% when corrective and substantive feedback were given, respectively. In contrast, pretesting led to significantly lower intrusion rates in all three studies (13%, 8%, and 2%, respectively) even though it was just as effective in yielding equivalent levels of overall final test performance. As such, our results suggest that if educators want to implement True/False practice tests to enhance learning, but are concerned about negative suggestion effects, pretesting may be the safer pedagogical choice. It provides a comparable benefit with a significantly lower risk of instilling false information. In fact, we failed to find any evidence of a negative suggestion effect in the pretesting condition of Experiment 3. When participants were given substantive feedback, they were no more likely to recall the false information on the final test than they would have been had they not been exposed to that false information at all. Of course, it is possible that following a more extended delay, a negative suggestion effect would re-emerge, similar to what has been observed in previous work on the sleeper effect in memory (Jacoby et al., 1989).

A few important limitations are worth noting, as they provide important context for our findings and suggest potential directions for future research. First, our participants consisted of undergraduate students at a university, and it remains to be seen whether the same patterns of intrusions would be observed in other populations, such as K-12 students in classroom settings. Second, our materials were limited to two short educational passages. The mechanisms may differ with more complex materials or as a function of prior knowledge or how related the false information is to the true information. Third, we encourage future research to examine the consequences of True/False testing following longer retention intervals, as the effects observed after a short delay may not necessarily be observed after a longer delay. Finally, True/False are typically not used in isolation. Rather, they are embedded within tests consisting of other kinds of items (e.g., multiple choice), and how participants respond and learn from True/False tests may differ as a function of such context.

In conclusion, the present research adds a new dimension to the discussion of the relative benefits of pretesting and posttesting. Although both pretesting and posttesting have the power to enhance final test performance (observed here in the context of True/False test-taking), posttesting appears to be most vulnerable to the negative suggestion effect. Investigating more precisely when and why this is the case represents a critical area for future theoretical and applied research in the science of learning.

## Data Availability

The raw data supporting the conclusions of this article will be made available by the authors on request.

## Appendix A

**Passage: Joan of Arc**				
	**True Item**	**False Item**	**Cued Recall Question**	**Substantive Feedback (Exp 3 Only)**
1	Before Joan of Arc convinced the embattled crown prince Charles of Valois to allow her to lead an army, she had no military experience.	Before Joan of Arc convinced the embattled crown prince Charles of Valois to allow her to lead an army, she had 5 years of military experience.	Before Joan of Arc convinced the embattled crown prince Charles of Valois to allow her to lead an army, how much military experience did she have? ________________.	She had *no military experience*.
2	In 1920, Joan of Arc was officially canonized as a saint.	In 1920, Joan of Arc was officially recognized on a list of famous female warriors.	In 1920, Joan of Arc was officially ________________.	She was *canonized*.
3	Joan of Arc was born around the year 1412.	Joan of Arc was born around the year 1300	Around what year was Joan of Arc born? __________________________.	She was born around *1412*.
4	Joan of Arc’s father worked as a farmer.	Joan of Arc’s father worked as a tailor.	Her father worked as a ___________________________.	He worked as a *farmer*.
5	Joan’s mother instilled in her a deep love for the Catholic Church.	Joan’s mother instilled in her a deep love for books and scholarly pursuits.	Joan’s mother instilled in her a deep love for ___________________.	She instilled a love for *the church*.
6	In 1420, when the French crown prince was disinherited, King Henry V was made ruler of England and France.	In 1420, when the French crown prince was disinherited, King Edward was made ruler of England and France.	In 1420, when the French crown prince was disinherited, ________________________ was made ruler of England and France.	King *Henry V* was made ruler.
7	Joan heard voices, which she believed were coming from God.	Joan heard voices, which she believed were coming from her deceased uncle.	Where did Joan believe the voices she heard were coming from?	She believed they were coming from *God*.
8	Joan went to court after her father tried to arrange a marriage for her.	Joan went to court after her father began to physically abuse her.	Joan went to court after her father ____________________.	He tried to *arrange a marriage* for her.
9	As part of her mission, Joan took a vow of chastity.	As part of her mission, Joan took a vow of silence.	As part of her mission, Joan took a vow of __________________________.	She took a vow of *chastity*.
10	Joan dressed in men’s clothes to make the 11-day journey across enemy territory to Chinon, the site of the crown prince’s palace.	Joan dressed in a black dress to make the 11-day journey across enemy territory to Chinon, site of the crown prince’s palace.	Joan dressed in __________________ to make the 11-day journey across enemy territory to Chinon, site of the crown prince’s palace.	She dressed in *men’s clothes*.
11	Joan promised Charles she would see him crowned king at Reims, the traditional site of French royal investiture.	Joan promised Charles she would see him crowned king at Versailles, the traditional site of French royal investiture.	Joan promised Charles she would see him crowned king at ______________________, the traditional site of French royal investiture.	Charles would be crowned at *Reims*.
12	After Joan’s early victory, she and her followers escorted Charles across enemy territory, enabling him to be coronated in July 1429.	After Joan’s early victory, she and her followers escorted Charles across enemy territory, enabling him to be freed from enemy capture in July 1429.	After Joan’s early victory, she and her followers escorted Charles across enemy territory, enabling him to be ____________________ in July 1429.	Charles was *coronated* at that time.
13	In the spring of 1430, the king ordered Joan to confront an assault on Compiégne carried out by the Anglo-Burgundians.	In the spring of 1430, the king ordered Joan to confront an assault on Compiégne carried out by the Austrians.	In the spring of 1430, the king ordered Joan to confront an assault on Compiégne carried out by the ______________________.	The assault was carried out by the *Anglo-Burgundians*.
14	When Joan was captured, she was forced to answer some 70 charges against her, including witchcraft, heresy, and dressing like a man.	When Joan was captured, she was forced to answer some 15 charges against her, including witchcraft, heresy, and dressing like a man.	When Joan was captured, she was forced to answer some _______ (#) charges against her, including witchcraft, heresy, and dressing like a man.	She had to answer *70* charges.
15	After a year in captivity and under threat of death, Joan relented and signed a confession saying she had never received divine guidance.	After a year in captivity and under threat of death, Joan relented and signed a confession saying she had captured and killed soldiers.	After a year in captivity and under threat of death, Joan relented and signed a confession saying ___________________________.	She signed a confession saying that she *did not receive divine guidance*.
16	Joan of Arc was 19 years old when she was burned at the stake.	Joan of Arc was 25 years old when she was burned at the stake.	How old was Joan when she was burned at the stake? ____________________	She was *nineteen*.

**Passage: Machu Picchu**				
	**True Item**	**False Item**	**Cued Recall Question**	**Substantive Feedback (Exp 3 Only)**
1	Machu Picchu is tucked away in the rocky countryside northwest of Cuzco, Peru.	Machu Picchu is tucked away in the rocky countryside northwest of Nazca, Peru.	Machu Picchu is tucked away in the rocky countryside northwest of ________________, Peru.	It is near *Cuzco*, *Peru*.
2	In the 16th century, the civilization that created Machu Picchu was nearly wiped out by Spanish Invaders.	In the 16th century, the civilization that created Machu Picchu was nearly wiped out by famine.	In the 16th century, the civilization that created Machu Picchu was nearly wiped out by ____________________.	It was nearly wiped out by *Spanish Invaders*.
3	Hiram Bingham is credited with discovering Machu Picchu in 1911.	Howard Carter is credited with discovering Machu Picchu in 1911.	____________________ is credited with discovering Machu Picchu in 1911.	*Hiram Bingham* is credited with this discovery.
4	The site stretches over an impressive 5-mile distance, featuring more than 3000 stone steps.	The site stretches over an impressive 5-mile distance, featuring more than 3000 buildings.	The site stretches over an impressive 5-mile distance, featuring more than 3000 __________________________.	It features more than 3000 *stone steps*.
5	It was abandoned an estimated 100 years after its construction.	It was abandoned an estimated 1000 years after its construction.	It was abandoned an estimated _____________________ years after its construction.	It was abandoned after *100 years*.
6	One theory of why Machu Picchu was deserted was that there was a smallpox epidemic.	One theory of why Machu Picchu was deserted was that there was a Malaria epidemic.	One theory of why Machu Picchu was deserted was that there was a/n _________________________ epidemic.	The theory states that there was a *smallpox epidemic*.
7	When the archaeologist credited with finding Machu Picchu arrived, he was actually trying to find Vilcabamba, the last Inca stronghold.	When the archaeologist credited with finding Machu Picchu arrived, he was actually trying to find Saqsaywaman, the first capital of Peru.	When the archaeologist credited with finding Machu Picchu arrived, he was actually trying to find ________________________________.	He was trying to find *Vilcabamba*, *the last Inca stronghold*.
8	The name Machu Picchu means Old Peak in the native Quechua language.	The name Machu Picchu means Green Tree in the native Quechua language.	The name Machu Picchu means ________________ in the native Quechua language.	The name means *Old Peak*.
9	The person who helped the archaeologist find Machu Picchu was 11 years old.	The person who helped the archaeologist find Machu Picchu was 80 years old.	How old was the person who helped the archaeologist find the site?	He was *11*.
10	When the excavated artifacts were brought to Yale University, a custody dispute was ignited.	When the excavated artifacts were brought to Columbia University, a custody dispute was ignited.	When the excavated artifacts were brought to _____________________ University, a custody dispute was ignited.	The artifacts were brought to *Yale*.
11	The artifacts of Machu Picchu were finally returned to Peru under Barack Obama.	The artifacts of Machu Picchu were finally returned to Peru under Bill Clinton.	Under which president were the artifacts finally returned to Peru?	They were returned under *Barack Obama*.
12	In the midst of a tropical mountain forest on the eastern slopes of the Andes mountains, Machu Picchu blends seamlessly into its natural setting.	In the midst of a tropical mountain forest on the eastern slopes of the Cordillera mountains, Machu Picchu blends seamlessly into its natural setting.	In the midst of a tropical mountain forest on the eastern slopes of the ___________________________, Machu Picchu blends seamlessly into its natural setting.	Machu Picchu is along the slopes of the *Andes* Mountains.
13	Its central buildings are prime examples of a masonry technique mastered by the Incas in which stones fit together without mortar.	Its central buildings are prime examples of a masonry technique mastered by the Incas in which stones were cut in interesting shapes.	Its central buildings are prime examples of a masonry technique mastered by the Incas in which stones _______________________________.	The stones were cut *to fit without mortar*.
14	The Intihuatana stone, is a sculpted granite rock that is believed to have functioned as a solar clock.	The Intihuatana stone, is a sculpted granite rock that is believed to have functioned as an idol for worshipping a god.	The Intihuatana stone, is a sculpted granite rock that is believed to have functioned as a ________________________.	It is believed to have functioned as a *solar clock or calendar*.
15	In 2007, Machu Picchu was designated as one of the new 7 wonders of the world.	In 2007, Machu Picchu was designated as one of the new sacred lands of Peru.	In 2007, Machu Picchu was designated as one of the new _______________________.	It was designated as one of the new 7 world wonders.
16	Machu Picchu is the most famous ruin in South America, welcoming hundreds of thousands of people per year.	Machu Picchu is the most famous ruin in South America, welcoming around ten thousand people per year.	Machu Picchu is the most famous ruin in South America, welcoming ____________________ (#) people per year.	The site welcomes *hundreds of thousands* of people per year
